# The Role of CD56 as an Immunophenotypic Marker in the Clinical Course of Multiple Myeloma

**DOI:** 10.3390/jcm15093492

**Published:** 2026-05-02

**Authors:** Azat Karabekov, Vadim Kemaikin, Zhandos Burkitbayev, Gulnur Zhakhina, Aigerim Sipenova, Inna Berger, Ulbolsyn Orumbayeva, Zhuldyz Iskakova, Rose Ibragimova, Nazym Temir, Gulnur Mamyr, Olga Kolesnikova, Fariza Shokubaeva, Akbota Tursyn, Meiramgul Yussupova, Aidana Shalkarbekova, Ayagul Ainabay, Alexandr Kolesnev

**Affiliations:** 1 Department of Oncohematology and Bone Marrow Transplantation, National Research Oncology Center LLP, Astana 010000, Kazakhstan; azat_32@mail.ru (A.K.); rozaibragimova31@gmail.com (R.I.); fariza_shokubaeva@mail.ru (F.S.); akbota-tursyn@mail.ru (A.T.); yussupovam@gmail.com (M.Y.); ainabaya@mail.ru (A.A.);; 2 National Research Oncology Center LLP, Astana 010000, Kazakhstan; 3 Department of Medicine, Nazarbayev University School of Medicine, Astana 010000, Kazakhstan; gulnur.zhakhina@nu.edu.kz; 4 Science Management Department, National Research Oncology Center LLP, Astana 010000, Kazakhstan; 5 Department of Oncohematology and Bone Marrow Transplantation, Republican Specialized Scientific and Practical Center of Hematology, Tashkent 100059, Uzbekistan; 6 University Medical Center CF, Astana 010000, Kazakhstan

**Keywords:** multiple myeloma, CD56 expression, induction therapy response, autologous stem cell transplantation, prognostic factors

## Abstract

**Background:** CD56 expression has been proposed as a prognostic and predictive biomarker in multiple myeloma. However, its clinical relevance in the context of modern induction therapy and autologous stem cell transplantation (ASCT) remains controversial. **Methods:** The researchers studied individuals with newly diagnosed multiple myeloma. CD56 expression was assessed by flow cytometry at diagnosis. Assessment was performed to determine patients’ responses to induction therapy and to measure their progression-free and overall survival rates. **Results:** The study included 88 participants, of whom 68 (77%) were CD56-positive, and 20 (23%) were CD56-negative. The study results showed that CD56(−) patients developed plasmacytomas at a 70% rate, while CD56(+) patients had a 46% rate (*p* = 0.055). A similar pattern was observed for extramedullary lesions (*p* = 0.006). The induction response rate was lower in CD56-negative patients than in CD56-positive patients (65% vs. 85%, *p* = 0.043). Patients who did not experience relapse received more CD34^+^ cells (11.9 ± 5.71 vs. 9.29 ± 2.57 × 10^6^/kg, *p* = 0.012) and had higher post-transplant response rates (91% vs. 63%, *p* = 0.002). The patients who received induction treatment before their disease showed better survival outcomes than patients who did not respond to treatment (90.2% vs. 87.1%, *p* = 0.029) and patients who did not experience relapse (92.7% vs. 85.0%, *p* = 0.022). CD56 status did not affect survival outcomes. **Conclusions:** CD56 expression is associated with disease burden and response to induction therapy in multiple myeloma, supporting its role as an early disease-modifying factor. However, its prognostic value appears limited in patients receiving high-dose chemotherapy with ASCT, suggesting that intensive treatment may mitigate the adverse impact of CD56 negativity.

## 1. Introduction

Multiple myeloma (MM) is a clonal plasma cell malignancy characterized by marked biological heterogeneity, diverse clinical manifestations, and substantial variability in treatment response and outcomes [1]. Over the past decade, major therapeutic advances, including proteasome inhibitors, immunomodulatory agents, and monoclonal antibodies, have significantly improved response rates and survival. Nevertheless, MM remains an incurable disease, and most patients ultimately experience disease relapse [2]. Autologous hematopoietic stem cell transplantation (auto-HSCT) continues to represent a cornerstone of therapy for transplant-eligible patients, providing deeper responses and prolonged disease control. However, post-transplant outcomes vary considerably among patients, highlighting the need for improved prognostic tools to better predict treatment response and long-term outcomes [3].

Current prognostic models, including the International Staging System (ISS) and the Revised ISS (R-ISS), integrate clinical parameters and high-risk cytogenetic abnormalities to stratify patients with MM [1]. While these models offer important prognostic guidance, they do not fully capture the biological complexity of the disease. Immunophenotypic characteristics of malignant plasma cells, which may reflect tumor aggressiveness, interactions with the bone marrow microenvironment, and the potential for disease dissemination, are not incorporated into standard risk assessment frameworks [4].

In real-world clinical practice in Kazakhstan, approximately 60% of patients are still stratified using only the Durie–Salmon staging system. As a result, during the analysis of baseline data, we encountered important limitations, as modern prognostic scoring systems (ISS, R-ISS) and cytogenetic risk assessment are not consistently available for a substantial proportion of patients. Consequently, molecular and genetic diagnostic methods remain inaccessible for many patients in Kazakhstan, which prevented us from adequately applying these established prognostic tools. Therefore, our analysis relied on clinical parameters that are objectively available in routine practice and guide treatment decisions regarding induction therapy. Missing data were not imputed, and analyses were conducted using only the data available for each patient.

Daratumumab recently become available as a first-line treatment option in the country, which makes the identification of accessible biomarkers particularly relevant. Such markers may help guide the choice between three-drug and four-drug induction regimens. Given the limitations of healthcare resources, it is not feasible to provide four-drug therapy to all patients. Therefore, there is a clear need for readily available markers that can support rational and individualized treatment decisions in routine clinical practice. Multiparameter flow cytometry is an essential component of MM diagnostics, enabling confirmation of diagnosis, assessment of tumor burden, and detection of measurable residual disease (MRD) [4]. In addition, this technique allows detailed characterization of plasma cell surface antigen expression. Among these markers, CD56 (neural cell adhesion molecule, NCAM) has been extensively studied. CD56 is aberrantly expressed on clonal plasma cells in a substantial proportion of MM cases and plays a critical role in mediating adhesion to bone marrow stromal cells. Loss of CD56 expression has been associated with impaired bone marrow retention, increased tumor cell dissemination, and a higher likelihood of extramedullary disease, suggesting a more aggressive disease phenotype [5,6]. Several contemporary studies have reported inferior survival outcomes in patients with CD56-negative MM; however, results across cohorts remain inconsistent, likely reflecting differences in study populations, treatment strategies, and follow-up duration [7].

Multiple myeloma pathogenesis is an intricate dynamic process involving the interaction between mycoid plasma cells and the bone marrow microenvironment. An increasing number of adhesion molecules, immune escape mechanisms and stromal molecules have been characterized that may affect survival, migration and drug-resistance of the mycoid plasma cells [8]. Importantly, as the disease progresses, the mycoid plasma cells may undergo changes which render them less dependent on the bone marrow microenvironment and therefore more prone to circulate to extra-medullary sites [9,10], which may contribute to disease progression and the evolution towards more aggressive disease phases.

Despite these advances, the relationship between plasma cell immunophenotype at diagnosis, particularly CD56 expression, and post-transplant immune recovery, relapse risk, and survival outcomes remains insufficiently defined [11]. Therefore, the present study aimed to evaluate the impact of CD56 expression on the effectiveness of induction therapy and overall survival in patients with multiple myeloma. By integrating immunophenotypic and immunological parameters, this study seeks to refine prognostic stratification and support the development of individualized, risk-adapted therapeutic strategies for patients with MM. In the last few years, monoclonal antibodies, bispecific antibodies and cellular therapies have dramatically changed the treatment of multiple myeloma and it is likely that the impact of some baseline biomarkers has changed, including immunophenotypic markers such as CD56 expression [12].

## 2. Materials and Methods

### 2.1. Study Design and Population

This retrospective, single-center study was conducted at the National Research Oncology Center (Astana, Republic of Kazakhstan). The study included patients with newly diagnosed multiple myeloma who initiated treatment between 2023 and 2025. The study cohort included consecutive patients meeting the inclusion criteria during the study period. A total of 88 patients were included in the final analysis. The diagnosis of multiple myeloma was established according to the International Myeloma Working Group (IMWG) criteria [13]. Clinical, laboratory, immunophenotypic, treatment-related, and outcome data were retrospectively collected from electronic medical records.

Missing data were handled using a complete-case approach where appropriate. Patients with more than 50% missing variables were excluded from the analysis. For variables with up to 40% missing data, multiple imputation was performed using age, sex, and CD56 status as predictor variables. No variables had more than 40% missing data, as missing information was minimized through additional data retrieval from archived medical records.

### 2.2. Immunophenotypic Analysis

Immunophenotyping of bone marrow plasma cells was performed by multicolor flow cytometry at the University Medical Center (UMC) in Astana, Republic of Kazakhstan. Standardized EuroFlow antibody panels were used according to established protocols to identify and characterize plasma cells. Clonal plasma cells were identified using backbone markers in combination with aberrant antigen expression patterns, enabling reliable discrimination of malignant plasma cells from their normal counterparts, in line with international consensus recommendations [14,15].

Immunophenotyping was performed as part of a routine diagnostic workup and is available in most regions of Kazakhstan. Bone marrow samples were analyzed by identifying plasma cells based on bright expression of CD38 and CD138, along with aberrant immunophenotypic features and immunoglobulin light chain restriction to define the clonal population.

To ensure that CD56 expression was evaluated specifically on clonal plasma cells, doublets, cellular debris, CD3-positive T lymphocytes, and CD16/CD56-positive NK cell populations were excluded during the analysis. To ensure adequate biological and prognostic relevance of the immunophenotypic analysis, only patients with a bone marrow clonal plasma cell infiltration of at least 10% were included. This threshold was selected because immunophenotypic features are considered less stable and of limited prognostic value in cases with minimal plasma cell involvement (1–2%) [16,17]. CD56 expression was assessed on clonal plasma cells using flow cytometry and classified qualitatively as positive or negative based on the presence or absence of detectable expression, in accordance with routine laboratory practice. CD56 expression was analyzed as a binary variable for clinical interpretability; however, this approach may oversimplify a biologically continuous parameter and represents a limitation of the study.

### 2.3. Treatment and Response Assessment

All patients received induction therapy with the VCD regimen, consisting of bortezomib, cyclophosphamide, and dexamethasone.

As previously described, the use of the VCD regimen was justified by the fact that this treatment approach remains the most widely used option in routine clinical practice in Kazakhstan. To ensure the formation of a makcиmaльho homogeneous cohort, patients who received alternative induction regimens were not included.

Two evaluation timepoints were defined in the study. The first corresponded to the end of the second cycle of induction therapy, at which point response assessment was limited to serum and urine immunofixation. The second timepoint was after completion of four cycles, when full restaging was performed according to IMWG criteria. Response to induction therapy was assessed according to the IMWG response criteria [13]. For analytical purposes, treatment response was dichotomized into response (partial response or better, including partial response, very good partial response, complete response, and stringent complete response) and no response (stable disease, progressive disease, or relapse). Overall survival was defined as the time from diagnosis to death from any cause or last follow-up. Patients without documented events were censored at the date of last follow-up. Due to sample size limitations, treatment response was dichotomized; however, depth of response and minimal residual disease were not assessed

In this study, all patients underwent autologous hematopoietic stem cell transplantation (auto-HSCT). The conditioning regimen consisted of melphalan at a dose of 200 mg/m^2^. Response assessment was performed at day +100 post-transplant according to IMWG criteria.

Patients who maintained or deepened their response were subsequently transitioned to maintenance therapy with the immunomodulatory agent lenalidomide, which was continued until disease progression or the development of unacceptable toxicity.

### 2.4. Statistical Analysis

Statistical analysis was performed using Stata/MP version 19.5 (StataCorp LLC, College Station, TX, USA). Continuous variables were summarized as medians with interquartile ranges, while categorical variables were reported as absolute numbers and percentages. Comparisons between CD56-negative and CD56-positive groups were conducted using the Mann–Whitney U test for continuous variables and the chi-square test or Fisher’s exact test, as appropriate, for categorical variables. Survival outcomes were analyzed using the Kaplan–Meier method and Cox regression, and differences between groups were assessed using the log-rank test. Survival outcomes were analyzed using the Kaplan–Meier method and exploratory Cox regression analysis. Multivariable modeling was not performed due to the limited sample size and insufficient number of events, which did not allow for a stable and reliable model. Attempts to construct multivariable models resulted in poor model fit and unstable estimates, with loss of statistical significance across variables; therefore, such analyses were not considered appropriate for interpretation. A two-sided *p*-value of less than 0.05 was considered statistically significant.

## 3. Results

This study included 88 patients, of whom 68 (77%) were CD56-positive and 20 (23%) were CD56-negative. Table 1 represents baseline clinical and laboratory characteristics of patients. The median age was 56 years (IQR: 49–61 years) and was not affected by CD56 status (*p* = 0.114). Females accounted for 52% of the total patients (40% in the CD56(−) and 56% in the CD56(+) group; *p* = 0.211). The distribution of blood paraprotein types was similar across groups (*p* = 0.395). Paraproteins were predominantly of the IgG type (74%), light chains were involved in 14% of cases, and 11% consisted of IgA, whilst only 1% were IgM. No significant difference was found in the presence of Bence–Jones proteins between CD56(−) and CD56(+) (55% vs. 60%, *p* = 0.672).

Plasma cells infiltrating the bone marrow were found at significantly different levels by flow cytometry, based on CD56 status. Individuals in the CD56(−) group had a significantly higher proportion of plasma cells in their plasma than in the CD56(+) group (26% vs. 13%, *p* < 0.001). However, the plasma cell percentage, as determined by bone marrow histological examination, did not differ significantly between the two groups. Regarding disease manifestations, 70% of CD56(−) patients had a plasmacytoma, which occurred in 46% of CD56(+) patients, approaching statistical significance (*p* = 0.055). In comparison, extra-medullary lesions were observed in 25% of CD56-negative cases, whereas they were observed in just 3% of CD56-positive cases (*p* = 0.006). The disease response to the first treatment phase varied significantly by CD56 status: CD56(−) patients had a lower response rate than CD56(+) patients (65% vs. 85%, *p* = 0.043).

After transplantation, 60% had no recurrence, whereas 40% did (Table 2). Both age and gender distributions were similar across the groups (both *p* > 0.05). In addition, there was no significant difference in CD56 expression between patients in remission and those with disease relapse (*p* = 0.981).

In this study, an induction response was observed in 81% of patients; however, there was a non-significant trend toward a higher response rate in the non-relapse group (87% vs. 71%, *p* = 0.074). Patients without a recurrence received a significantly greater dose of CD34^+^ cells (11.9 ± 5.71 vs. 9.29 ± 2.57 × 10^6^/kg, *p* = 0.012) and achieved a higher response rate following transplantation (91% vs. 63%, *p* = 0.002). Both mucositis rates and infection incidence were similar between the groups. The median engraftment time was longer in patients who relapsed than in those who did not (19 days vs. 17 days, *p* = 0.057).

**Figure 1:**
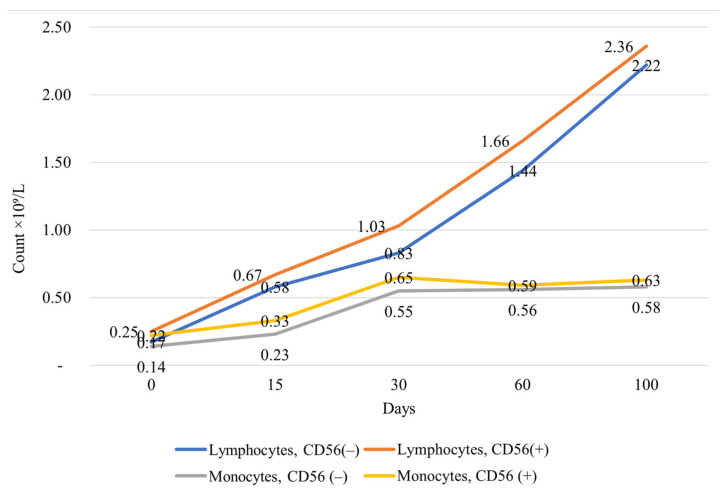
Post-transplant dynamics of lymphocyte and monocyte counts stratified by CD56 expression.

Figure 1 demonstrates how lymphocyte and absolute monocyte counts (×10^9^/L) changed on days 0, 15, 30, 60, and 100 after the bone marrow transplantation relative to CD56 expression. Both the CD56-negative and the CD56-positive patients showed an increase in lymphocyte counts from 0.17 to 2.22 × 10^9^/L in the former and from 0.25 to 2.36 × 10^9^/L in the latter between day 0 and day 100. The number of monocytes first rose and then leveled out in both the CD56(+) and CD56(−) groups, reaching 0.14 to 0.58 × 10^9^/L in CD56-negative patients and 0.22 to 0.63 × 10^9^/L in CD56-positive patients by the 100th day. The highest count of 0.65 × 10^9^/L was recorded at day thirty. Over the observation period, the lymphocyte and monocyte counts were higher in the CD56-positive patients. Still, this difference was not statistically significant compared with CD56-negative patients.

**Figure 2:**
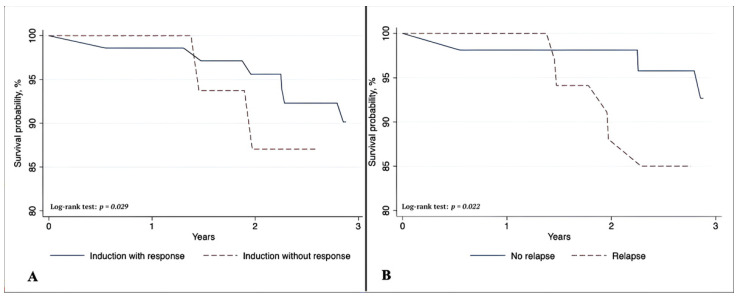
Overall survival according to induction response (**A**) and relapse status (**B**).

Kaplan–Meier curves in Figure 2 illustrate overall survival over three years, stratified by induction response (A) and relapse status (B). Patients achieving an induction response had higher survival than non-responders, with a 90.2% versus 87.1% survival rate (log-rank *p* = 0.029). Similarly, survival differed by relapse status. Patients without relapse demonstrated 92.7% 3-year survival, compared with 85.0% in the relapse group (log-rank *p* = 0.022). Corresponding analyses of overall survival (OS) and progression–free survival (PFS) is provided in Supplementary Figure S1A,B. Given the relatively small sample size, survival estimates should be interpreted with caution, particularly at later time points where the number of patients at risk may be limited.

The associations between overall survival and baseline, disease-related, and BMT-related variables were examined using the Cox regression model (Table 3). Most of the analyzed variables were not significantly correlated with the outcome. The patients with plasmacytoma had a higher risk of an unfavorable outcome. Their risk of death was 3.24 times higher (*p* = 0.042) than for those without this complication. In contrast, those with extramedullary lesions or renal failure did not have a significantly higher risk of death. Response to induction treatment has also been shown to be a significant prognostic factor. Non-responders to the therapy had a higher risk of death than those who responded to the treatment (*p* = 0.037; HR = 2.88, 95% CI 1.07–7.81).

## 4. Discussion

The research investigated the presence of the CD56 surface marker on cancerous plasma cells from newly diagnosed multiple myeloma patients who initiated conventional induction therapy and contemporary supportive treatments at first diagnosis. The research results provided vital information on the effects of CD56 on biological systems and clinical outcomes, which scientists used to better understand how immunophenotypic markers affect prognosis more than current staging systems do.

According to data from the national electronic cancer registry, more than 200 new cases of multiple myeloma are registered annually in Kazakhstan. Given the limited use of R-ISS/R2-ISS staging systems in routine practice, selecting the most appropriate induction chemotherapy regimen remains challenging. The universal application of four-drug induction regimens is associated with substantial costs and is not feasible within the current healthcare resource constraints in Kazakhstan. As a result, there is a relevant clinical need to optimize induction therapy selection under conditions of limited access to molecular and genetic testing in patients with newly diagnosed multiple myeloma. In this context, CD56 was not considered as a replacement for established molecular and genetic risk stratification. Rather, our aim was to evaluate whether this marker could serve as an additional tool for initial risk assessment, particularly one that is widely accessible across healthcare settings. The association between CD56-negative cells and unfavorable disease outcomes suggests that CD56 expression marks vital biological functions performed by plasma cells. The neural cell adhesion molecule CD56 functions as a mediator that enables plasma cells to connect with their surrounding bone marrow environment [8]. The loss of CD56 surface markers leads to decreased bone marrow cell retention, increased cell movement, and a greater likelihood of cancer cells leaving the bone marrow [18]. Findings from previous studies indicate that CD56-negative multiple myeloma cells are more aggressive. Several studies of newly diagnosed multiple myeloma patients showed that CD56-negative ones developed more severe medical conditions, which included higher ISS stage, worse ECOG performance status, and elevated β2-microglobulin and creatinine levels [19,20,21,22]. The lack of CD56 expression in patients was associated with shorter survival times, affecting both disease progression and overall lifespan. In our cohort, CD56 negativity was associated with adverse disease features and lower response rates; however, its independent impact on survival could not be reliably established. However, we did not find a significant association between CD56 status and overall survival in multivariate analysis.

The association between CD56-negative cases and shorter survival can be explained by its biological role in plasma cell-bone marrow interactions. These findings can be interpreted within the framework of tumor–microenvironment interactions, where loss of adhesion molecules such as CD56 may reflect reduced dependence on the bone marrow niche and increased dissemination potential. This evolutionary trajectory highlights the role of the bone marrow microenvironment not only in disease progression but also in shaping therapeutic resistance [9]. Disruption of tumor–microenvironment interactions may therefore represent a potential therapeutic strategy, particularly in patients with more aggressive or disseminated disease phenotypes. The absence of CD56 expression may reflect a more aggressive plasma cell phenotype, with reduced attachment to bone marrow cells, increased movement between sites, and a higher risk of spreading outside the bone marrow, leading to worse patient survival. For example, this study found that CD56(−) patients had a significantly higher frequency of extramedullary lesions and plasmacytoma than CD56(+) patients. A similar pattern was found in research from China [16], Turkey [23], and America [24,25]. The results of the current study confirm this biological model, as CD56-negative disease is characterized by both increased tumor size and widespread disease, thereby validating CD56 as a disease behavior indicator rather than a simple diagnostic tool.

This research found that CD56 expression was also associated with the response to induction therapy, suggesting that immunophenotypic features at diagnosis may influence treatment sensitivity. Other studies have reported conflicting results on the association between CD56 status and induction response, which may stem from differences in treatment methods, study participants, and testing methods [12,26]. For example, several cross-sectional studies have reported that patients with CD56-negative multiple myeloma demonstrate a lower response to frontline therapy. This reduced responsiveness has been observed across different treatment backbones, including both bortezomib- and daratumumab-based regimens, suggesting that the adverse impact of CD56 loss on early treatment response may be independent of the specific induction strategy [20,27]. As stated before, patients in the current study received VCD-based induction therapy comprising bortezomib, cyclophosphamide, and dexamethasone. On the other hand, some studies show little or no association between CD56 status and frontline treatment. Several cohort studies report no significant differences in treatment response or survival outcomes between CD56-negative and CD56-positive patients, particularly among those receiving high-dose therapy and autologous stem cell transplantation [28,29]. In addition, a meta-analysis shows that studies conducted at different times produced inconsistent results, suggesting that CD56 status is an inadequate single indicator for predicting treatment outcomes [30].

The expression of CD56 did not affect relapse rates or survival outcomes after transplantation, according to the current study. This finding suggests that while CD56 status may be informative at diagnosis and during initial treatment, it becomes less effective at predicting outcomes after patients receive high-dose chemotherapy with autologous stem cell transplantation. Other research showed similar patterns: multiple myeloma patients who receive high-dose chemotherapy with ASCT do not show differences in treatment outcomes based on CD56 expression levels [26,28,30]. The results of this study align with recent scientific findings, which show that post-transplant outcomes depend on how well patients respond to treatment, their immune system recovery, and transplant complications rather than their initial cancer characteristics. The observed association between higher CD34^+^ cell dose and lower relapse rates in this study should be interpreted with caution. Mobilization capacity may reflect underlying disease biology and depth of response to induction therapy, as patients achieving better disease control are more likely to mobilize stem cells more effectively [31,32]. Therefore, the relationship between CD34^+^ cell dose and outcomes may be confounded by treatment response rather than representing an independent prognostic factor. CD56 expression may represent an early disease-related characteristic influencing clinical presentation and treatment response rather than a definitive determinant of long-term survival.

This study has several important strengths. The research offers several key advantages that make it valuable. The research examines how CD56 markers affect treatment outcomes in multiple myeloma patients undergoing modern induction therapies by analyzing a specific patient group. The research examines CD56′s effects on patient outcomes and life expectancy after high-dose chemotherapy with autologous stem cell transplantation to establish its role as a disease management tool that extends beyond its impact on survival. The research findings supported previous studies, which showed both positive and insignificant results, thus confirming our study’s detailed and extensive findings.

On the other hand, several limitations need to be recognized. The retrospective design method creates two potential research problems: selection bias and unmeasured confounding factors. The research results from this single-site investigation do not apply to all patient groups because they were obtained from patients who received specific induction treatment without monoclonal antibodies. The study maintains a comparable cohort size to other immunophenotypic multiple myeloma research, but its limited number of events restricts its ability to perform powerful multivariable survival analyses, potentially leading missed small correlations. The association between demographic and clinical variables and CD56 expression was examined using binary methods, which may have limited the power of the reported results.

The findings should also be interpreted in the context of evolving treatment strategies, as the absence of antibody-based regimens may limit generalizability to current clinical practice. In addition, the predominantly univariate analytical approach without full adjustment for key prognostic variables limits the ability to draw causal inferences. The lack of detailed data on dose intensity and minimal residual disease further restricts the interpretation of treatment outcomes. Furthermore, the discrepancy observed between flow cytometry and morphological assessment of plasma cell infiltration was not formally investigated and may reflect either technical variability or underlying biological heterogeneity. Finally, given the relatively small sample size, survival estimates should be interpreted with caution, particularly at later time points, when the number of patients at risk may be limited. Therefore, these findings should be considered hypothesis-generating and require validation in larger, prospective studies.

## 5. Conclusions

This study suggests that immunophenotypic characteristics may provide additional information in the clinical evaluation of multiple myeloma to improve outcomes. The initial expression of CD56 markers correlates with disease characteristics and treatment responses, but multiple factors, including medical treatments and individual patient biology, also affect final outcomes. The research results indicate that scientists should conduct additional studies to develop prognostic models that use immunophenotypes to improve myeloma treatment for individual patients.

## Figures and Tables

**Table 1:** Baseline clinical and laboratory characteristics of study participants stratified by CD56 expression.

	Total (n = 88)	CD56		*p*-Value
		Negative (n = 20; 23%)	Positive (n = 68; 77%)	
Age, years (median [IQR])	56 [49–61]	51 [42–61]	57 [51–62]	0.114
Gender, n (%)				0.211
Female	46 (52)	8 (40)	38 (56)	
Male	42 (48)	12 (60)	30 (44)	
Blood paraprotein, n (%)				0.395
IgA	10 (11)	2 (10)	8 (12)	
IgG	65 (74)	13 (65)	52 (76)	
IgM	1 (1)	0	1 (2)	
Light-chain	12 (14)	5 (25)	7 (10)	
Bence–Jones protein, n (%)				0.672
Absent	36 (41)	9 (45)	27 (40)	
Present	52 (59)	11 (55)	41 (60)	
Lymphocytes, ×10^9^/L (median [IQR])	1.50 [1.12–2.07]	1.70 [1.24–1.98]	1.50 [1.10–2.09]	0.819
Monocytes, ×10^9^/L (median [IQR])	0.40 [0.25–0.60]	0.36 [0.25–0.59]	0.41 [0.25–0.60]	0.679
IgA, g/L (median [IQR])	0.27 [0.14–0.70]	0.18 [0.10–0.68]	0.30 [0.19–0.89]	0.313
IgG, g/L (median [IQR])	13.5 [5.57–44.9]	11.12 [4.98–18.3]	15.5 [5.91–53.2]	0.353
IgM, g/L (median [IQR])	0.25 [0.12–0.41]	0.13 [0.04–0.41]	0.26 [0.13–0.41]	0.201
Bone marrow plasma cells by flow cytometry (%), (median [IQR])	15 [10–24]	26 [16.5–35.5]	13 [7.8–18]	<0.001
Bone marrow plasma cells (morphology, %), (median [IQR])	24.6 [9.1–40.2]	30.9 [7.8–41.8]	23.9 [10.5–40]	0.673
Plasmacytoma, n (%)				0.055
Absent	43 (49)	6 (30)	37 (54)	
Present	45 (51)	14 (70)	31 (46)	
Extramedullary lesions, n (%)				0.006
Absent	81 (92)	15 (75)	66 (97)	
Present	7 (8)	5 (25)	2 (3)	
Renal failure				0.502
Yes	20 (23)	4 (20)	16 (24)	
No	68 (77)	16 (80)	52 (76)	
Induction response, n (%)				0.043
Response	71 (81)	13 (65)	58 (85)	
No response	17 (19)	7 (35)	10 (15)	

* IQR—interquartile range.

**Table 2:** Baseline characteristics and post-transplant outcomes of study participants stratified by relapse status.

	Total (n = 88)	Relapse		*p*-Value
		No (n = 53; 60%)	Yes (n = 35; 40%)	
Age, years (mean SD)	54 (8)	55 (9)	54 (8)	0.423
Gender, n (%)				0.897
Female	46 (52)	28 (53)	18 (51)	
Male	42 (48)	25 (47)	17 (49)	
CD56, n (%)				0.981
Negative	20 (23)	12 (23)	8 (23)	
Positive	68 (77)	41 (77)	27 (77)	
Induction response, n (%)				0.074
Response	71 (81)	46 (87)	25 (71)	
No response	17 (19)	7 (13)	10 (29)	
Number of infused CD34^+^ hematopoietic stem cells (×10^6^/kg), mean (SD)	10.9 (4.87)	11.9 (5.71)	9.29 (2.57)	0.012
BMT response, n (%)				0.002
Response	70 (80)	48 (91)	22 (63)	
No response	18 (20)	5 (9)	13 (37)	
Infectious complications after BMT, n (%)				0.264
Yes	62 (70)	35 (66)	27 (77)	
No	26 (30)	18 (34)	8 (23)	
Mucositis, n(%)				0.694
No	33 (37)	19 (36)	14 (40)	
Yes	55 (63)	34 (64)	21 (60)	
Engraftment, days (median [IQR])	18 [16–20]	17 [15–19]	19 [16–21]	0.057

* BMT—bone marrow transplantation.

**Table 3:** Unadjusted Cox proportional hazards analysis of factors associated with overall survival.

	Unadjusted Model	
	HR [95% CI]	*p*-Value
Age (years)	0.98 [0.92–1.04]	0.471
Gender [ref. female]		
Male	1.12 [0.42–2.99]	0.824
Bence–Jones protein [ref. absent]		
Absent		
Lymphocytes (×10^9^/L)	0.63 [0.32–1.25]	0.186
Monocytes (×10^9^/L)	0.22 [0.04–1.30]	0.095
Bone marrow plasma cells by flow cytometry (%)	1.00 [0.97–1.03]	0.855
Bone marrow plasma cells (morphology, %)	0.99 [0.97–1.02]	0.646
Plasmacytoma [ref. absent]		
Present	3.24 [1.04–10.1]	0.042
Extramedullary lesions [ref. absent]		
Present	3.04 [0.86–10.8]	0.086
Renal failure [ref. no]		
Yes	1.76 [0.61–5.08]	0.296
Induction response [ref. response]		
No response	2.88 [1.07–7.81]	0.037
CD56 [ref. negative]		
Positive	0.85 [0.27–2.65]	0.783
Number of infused CD34^+^ hematopoietic stem cells (×10^6^/kg)	0.93 [0.82–1.06]	0.271
BMT response [ref. response]		
No response	0.95 [0.293.00]	0.924
Infectious complications after BMT [ref. no]		
Yes	1.78 [0.50–6.31]	0.372
Mucositis [ref. no]		
Yes	1.04 [0.38–2.88]	0.940
Engraftment (days)	1.06 [0.96–1.16]	0.245

* HR—Hazard Ratio; * CI—Confidence interval.

## Data Availability

The data presented in this study are not publicly available due to privacy and ethical restrictions related to patient confidentiality.

## Supplementary Materials

The following supporting information can be downloaded at: https://www.mdpi.com/article/10.3390/jcm15093492/s1.

## References

[1] Rajkumar S.V. (2022). Multiple myeloma: 2022 update on diagnosis, risk stratification, and management. Am. J. Hematol..

[2] Kumar S.K., Callander N.S., Adekola K., Anderson L., Baljevic M., Campagnaro E., Castillo J.J., Chandler J.C., Costello C., Efebera Y. (2020). Multiple myeloma, version 3.2021, NCCN clinical practice guidelines in oncology. J. Natl. Compr. Cancer Netw..

[3] Cowan J., Green D.J., Kwok M., Lee S., Coffey D.G., Holmberg L.A., Tuazon S., Gopal A.K., Libby E.N. (2022). Diagnosis and management of multiple myeloma: A review. JAMA.

[4] Paiva B., Puig N., Cedena M.-T., Rosiñol L., Cordón L., Vidriales M.-B., Burgos L., Flores-Montero J., Sanoja-Flores L., Lopez-Anglada L. (2020). Measurable residual disease by next-generation flow cytometry in multiple myeloma. J. Clin. Oncol..

[5] Wang Y., Feng W., Liu P. (2020). Genotype–immunophenotype analysis reveals the immunogenomic subtype and prognosis of multiple myeloma. Carcinogenesis.

[6] Ismail N.H., Chi L.P., Yusoff N.R.M.N., Mohamed R., Hamzah R., Johan M.F., Hassan R., Yusoff S.M. (2024). Immunophenotypic Expression and its Association with Prognostic Factors, Clinical Stages, and Clinical Profiles in Newly Diagnosed Patients with Plasma Cell Myeloma: Insights from Two Tertiary Care Centers. Biomed. Res. Ther..

[7] Guo J., Su J., He Q., Li X., Zhao Y., Gu S., Fei C., Chang C. (2016). The prognostic impact of multiparameter flow cytometry immunophenotyping and cytogenetic aberrancies in patients with multiple myeloma. Hematology.

[8] Zerdan M.B., Nasr L., Kassab J., Saba L., Ghossein M., Yaghi M., Dominguez B., Chaulagain C.P. (2022). Adhesion molecules in multiple myeloma oncogenesis and targeted therapy. Int. J. Hematol. Oncol..

[9] Solimando A.G., Malerba E., Leone P., Prete M., Terragna C., Cavo M., Racanelli V. (2022). Drug resistance in multiple myeloma: Soldiers and weapons in the bone marrow niche. Front. Oncol..

[10] Fotiou D., Katodritou E. (2025). From Biology to Clinical Practice: The Bone Marrow Microenvironment in Multiple Myeloma. J. Clin. Med..

[11] Koumpis E., Tassi I., Malea T., Papathanasiou K., Papakonstantinou I., Serpanou A., Tsolas E., Kapsali E., Vassilakopoulos T.P., Papoudou-Bai A. (2021). CD56 expression in multiple myeloma: Correlation with poor prognostic markers but no effect on outcome. Pathol.-Res. Pract..

[12] Devasia A.J., Chari A., Lancman G. (2024). Bispecific antibodies in the treatment of multiple myeloma. Blood Cancer J..

[13] International Myeloma Working Group (2014). International Myeloma Working Group (IMWG) Criteria for the Diagnosis of Multiple Myeloma.

[14] Rawstron C., Orfao A., Beksac M., Bezdickova L., Brooimans R.A., Bumbea H., Dalva K., Fuhler G., Gratama J., Hose D. (2008). Report of the European Myeloma Network on multiparametric flow cytometry in multiple myeloma and related disorders. Haematologica.

[15] Flores-Montero J., Sanoja-Flores L., Paiva B., Puig N., García-Sánchez O., Böttcher S., van der Velden V.H.J., Pérez-Morán J.J., Vidriales M.B., García-Sanz R. (2017). Next Generation Flow for highly sensitive and standardized detection of minimal residual disease in multiple myeloma. Leukemia.

[16] Rajkumar S.V., Dimopoulos M.A., Palumbo A., Blade J., Merlini G., Mateos M.V., Kumar S., Hillengass J., Kastritis E., Richardson P. (2014). International Myeloma Working Group updated criteria for the diagnosis of multiple myeloma. Lancet Oncol..

[17] Leonardos D., Benetatos L., Apostolidou E., Koumpis E., Dova L., Kapsali E., Kotsianidis I., Hatzimichael E. (2025). Applications of multiparameter flow cytometry in the diagnosis, prognosis, and monitoring of multiple myeloma patients. Diseases.

[18] Ho M., Paruzzo L., Minehart J., Nabar N., Noll J.H., Luo T., Garfall A., Zanwar S. (2025). Extramedullary multiple myeloma: Challenges and opportunities. Curr. Oncol..

[19] Wang X.-X., Zhang L.-L., Wang T., Hou J.-X., Wang Z.-T., Qin H. (2023). Prognostic Value of CD56 Expression in Newly Diagnosed Multiple Myeloma Patients and Its Related Factors. Zhongguo Shi Yan Xue Ye Xue Za Zhi.

[20] Robinette J., Huric L., Dona K., Benson D., Cottini F. (2024). CD56 expression predicts response to Daratumumab-based regimens. Blood Cancer J..

[21] Al-Ahmed M.A., Al-Rubaie H.A. (2025). The role of CD319, CD117, CD28, CD49e, CD56, and CD44 expression as diagnostic and prognostic markers in multiple myeloma. Iraqi J. Hematol..

[22] Cottini F., Benson D. (2023). To be or not to be: The role of CD56 in multiple myeloma. Oncotarget.

[23] Güvenç B. (2025). CD56-Negative Conjunctival Solitary Extramedullary Plasmacytoma with Bence–Jones Lambda: Organ-Sparing Therapy and Durable Remission in a Young Adult. Hematol. Transfus. Cell Ther..

[24] Abdolahi M., Padilla O., Dave S.S., Thakkar D., Nejati R. (2025). Plasmablastic plasmacytoma followed by a plasmacytic plasma cell myeloma: Insights into discordant extramedullary transformation—A case report and literature review. J. Hematop..

[25] White K.D., Kerkvliet A.M., Jassim A.D. (2023). Aberrant Expression of CD56 in Metastatic Malignant Melanoma. Cutis.

[26] Geng C., Zhou H., Wang H., Li Y., Leng Y., Zhang Z., Jian Y., Yang G., Chen W. (2022). Newly diagnosed multiple myeloma patients with CD56 expression benefit more from autologous stem cell transplantation. BMC Cancer.

[27] Yoshida T., Ri M., Kinoshita S., Narita T., Totani H., Ashour R., Ito A., Kusumoto S., Ishida T., Komatsu H. (2018). Low expression of neural cell adhesion molecule, CD56, is associated with low efficacy of bortezomib plus dexamethasone therapy in multiple myeloma. PLoS ONE.

[28] Skerget M., Skopec B., Zadnik V., Zontar D., Podgornik H., Rebersek K., Furlan T., Cernelc P. (2018). CD56 expression is an important prognostic factor in multiple myeloma even with bortezomib induction. Acta Haematol..

[29] Hundemer M., Klein U., Hose D., Raab M.S., Cremer F.W., Jauch A., Benner A., Heiss C., Moos M., Ho A.D. (2007). Lack of CD56 expression on myeloma cells is not a marker for poor prognosis in patients treated by high-dose chemotherapy and is associated with translocation t (11; 14). Bone Marrow Transplant..

[30] Zhang L., Huang Y., Lin Y., Zhang A., Zou R., Xu H., Wang S. (2022). Prognostic significance of CD56 expression in patients with multiple myeloma: A meta-analysis. Hematology.

[31] Zhang T., Cheng X., Jin Y., Zhang R., Shen X., Chen L., Shi Q. (2026). Impact of CD34^+^ cell infusion dose on immune reconstitution and survival in multiple myeloma after autologous stem cell transplantation. Leuk. Lymphoma.

[32] Pasvolsky O., Marcoux C., Milton D.R., Pal B., Tanner M.R., Bashir Q., Srour S., Lee J., Saini N., Lin P. (2024). Optimal infused CD34^+^ cell dose in multiple myeloma patients undergoing upfront autologous hematopoietic stem cell transplantation. Blood Cancer J..

