# Supplementary figures and images for: The Role of CD56 as an Immunophenotypic Marker in the Clinical Course of Multiple Myeloma
Source: J Clin Med. 2026 May 2;15(9):3492. doi: 10.3390/jcm15093492 (PMC13164124; doi:10.3390/jcm15093492)

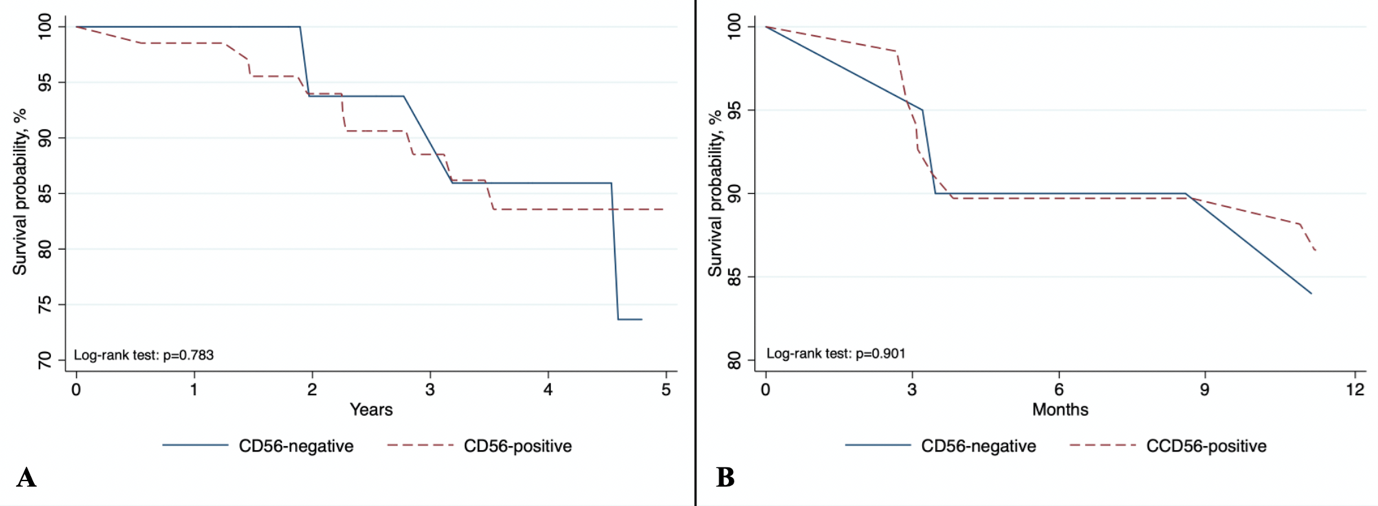

Supplement: Supplementary file 1 [file jcm-15-03492-s001.zip › jcm-4232029-supplementary.tiff]
